# The role of oxidative stress-mediated apoptosis in the pathogenesis of uric acid nephropathy

**DOI:** 10.1080/0886022X.2019.1633350

**Published:** 2019-07-04

**Authors:** Lijuan Yang, Baochao Chang, Yaling Guo, Xueping Wu, Lei Liu

**Affiliations:** a Department of Physiology, Bengbu Medical College, Bengbu, People’s Republic of China;; b Department of Nephrology, The First Affiliated Hospital of Bengbu Medical College, Bengbu, People’s Republic of China

**Keywords:** Uric acid nephropathy, oxidative stress, mitochondria, apoptosis, reduced glutathione

## Abstract

**Objective:** By copying the uric acid nephropathy rat model, the oxidative stress injury of mitochondria was caused in renal tubular epithelial cells and the relationship between the injury and the induction of cell apoptosis was identified.

**Methods:** All rats were randomly divided into NC (normal control, NC) group, HUA (high uric acid, HUA) group and GSH (reductive glutathione, GSH) group. The values were quantitatively tested in the kidney tissues, including 24-h urinary protein quantity, serum creatinine, blood uric acid, the MDA (malondialdehyde, MDA) and SOD (superoxide dismutase, SOD) oxidative stress indicators. The expression of p53, Bax and caspase-9/-3 were detected by immunoblotting. TUNEL assays were used to detect the apoptosis of renal tubular epithelial cells.

**Result:** In HUA and GSH groups, the 24-h urinary protein(24UTP), serum creatinine, and blood uric acid increased gradually with the increase of the replication cycle and the increase was significant compared to the NC group (*p* < .05). Compared to the NC group, MDA increased whereas SOD decreased. The expression of apoptotic proteins, such as p53, Bax, and caspase-9/-3 in the mitochondria was significantly different (*p* < .05). TUNEL assay revealed that the renal tubular epithelial cells in HUA group were largely apoptotic, whereas the GSH group improved significantly.

**Conclusion:** Mitochondria incurred the substantial damage due to being in a state of oxidative stress, which was the primary cause of apoptosis in the renal tubule epithelial cells. GSH exhibited the effective resistance to the influence of oxidative stress and can restore the damage in the renal tubular epithelial cells.

## 1. Preface

The incidence of hyperuricemia (HUA) is increasing year by year; this increase is related to diet, exercise, heredity, and other factors. Individuals with the chronic HUA are prone to hypertension, obesity, diabetes, cardiovascular and cerebrovascular diseases, and kidney injury [1]. It was reported that the oxidative stress of high uric acid levels directly caused kidney damage, which acted as an independent risk factor for the chronic kidney disease [2]. The prevention and treatment of HUA kidney injury has aroused extensive concern in clinicians. HUA’s mechanisms of kidney damage are diverse. The mitochondrial damage caused by oxidative stress has become a research hotspot in recent years. Researchers have found that the uric acid interfered with the rat renal tubular epithelial cells and may affect the mitochondrial structure and function. It would lead to the renal tubular epithelial cell apoptosis which was initiated by stimulating an oxidative stress reaction [3]. The mitochondria is the center of cell energy metabolism and is the primary place where oxidative phosphorylation occurs. Mitochondrial aerobic respiration can produce a large amount of ATP to maintain the energy necessarily needed in the life activities, and can generate a large number of oxygen free radicals through a series of biochemical reactions. When cells are in turbulent redox conditions, the oxygen free radicals are produced too much to be cleared; thus, the mitochondrial membrane lipid peroxidation occurs, the membrane fluidity is reduced, and the mitochondrial swelling occurs with the loss of select permeability which will lead to the apparent mitochondrial dysfunction and cell apoptosis [4,5]. Our previous research has found that the reduced glutathione can pass through oxidative stress resistant diaphragmatic muscle injury in a rat model of diabetes. Thus, whether reduced glutathione can improve the apoptosis of renal tubular epithelial cells in the uric acid nephropathy rats is needed to be further researched. Using the uric acid nephropathy in rats, the oxidative stress that caused the renal tubular epithelial cells mitochondrial damage was researched and the mechanisms of renal tubular epithelial cells apoptosis caused by mitochondrial injury was clarified. This study provided an experimental basis for the clinical prevention and treatment of uric acid nephropathy.

## 2. Materials and methods

### 2.1. The experimental materials

Sprague-Dawley (SD) rats and feed were purchased from Bengbu Medical College Animal Experimental Center. Yeast (purchased from Nanjing Institute of Bioengineering, CHN), adenine (purchased from Nanjing Institute of Bioengineering, CHN), a Mitochondrial extraction kit (purchased from Nanjing Jiancheng institute of Biological Engineering, CHN), MDA and SOD detection reagents (purchased from Nanjing Jiancheng Institute of Biological Engineering, CHN), detection of p53, Bax and caspase-9/-3 antibodies (purchased from Wuhan Seville Biotechnology Co., LTD., CHN), and a TUNEL kit (purchased from Roche, USA) were also used. This study was authorized by the Ethics Committee of Bengbu Medical College, batch no.2015.026.

### 2.2. The experimental method

#### 2.2.1. Replica animal model

A total of 30 healthy and clean male SD rats were divided into a normal control group (NC group), a HUA group, a reduced glutathione group (GSH group) and a normal feeding group. The normal control group was given ordinary feed for 1 week; the other two groups had yeast (0.6 g kg^−1^·D) and a joint adenine (0.1 g kg^−1^·D) added in the feed to fill their stomachs. At 1, 2, and 3 weeks, angular venous blood was extracted to examine changes in blood uric acid and was significantly higher than normal group, thus indicating success [6]. After the success of the building group raised again, the normal control group and uric acid group were fed chow; the GSH group, in addition to the ordinary feed, received intraperitoneal injections GSH (0.5 g kg^−1^·D) for 4 weeks. At the end of 4 weeks, rats in each group were sacrificed and their kidneys were taken for oxidative stress indicators, apoptotic protein expression and apoptosis detection.

#### 2.2.2. Collection and testing of blood and urine specimens

Quantitative levels of serum uric acid, creatinine and 24UTP were detected for 4 consecutive weeks after successful modeling. The blood samples were collected from the rats’ epicanthus vein, and the urine samples were collected in a urine collection cage. All samples were sent to the laboratory department of the First Affiliated Hospital of Bengbu Medical College for examination.

#### 2.2.3. MDA and SOD detection

We extracted the medullary tissue from the rat kidneys after killing the rats with anesthesia at the end of 4 weeks. The renal tissue was cut into pieces, and phenylmethylsulfonyl fluoride (PMSF) was added to produce a tissue homogenate. The homogenized tissue was vortexed at the highest available speed for 5 s. Then, the cell pellet was completely suspended and dispersed, the cytoplasmic protein extraction reagent was added, and the mixture was again vortexed at the highest speed for 5 s. After centrifugation for 5 min, the supernatant was transferred to a precooled plastic tube, determine the concentrations of MDA and SOD in strict accordance with the kit’s instructions.

#### 2.2.4. Mitochondrial extraction

Shortly after the rats were killed, the medullary tissue from the rat kidneys was removed using a Keygen mitochondrial extraction kit. A 2-g sample of kidney tissue was taken, and 1.5 mL Lysis Buffer was added and homogenized, after shearing. The medium buffer was composed of supernatant liquid product = 1:1 added to the reagent, covering the upper layer of the buffer. The washing buffer was added to the collected mitochondrial sediment and suspended again; then, the supernatant was discarded. The stored buffer overhang mitochondrial precipitate (100 pleigs/pistl) was used immediately or preserved at −80 absolute °C.

#### 2.2.5. Expression of p53, bax and caspase-9/-3 proteins in mitochondria with immunoblot

Based on the Keygen extracti on kit instructions, the renal tissue was cut into pieces, and the tissue was homogenized at the highest available speed for 5 s at 4 °C. Then, centrifuged the sediment in 200 μL mitochondrial extract (add DTT and protease inhibitors), and rapidly blended the mitochondrial proteins. The p53, Bax and caspase-9/-3 proteins were detected by immunoblot [7].

#### 2.2.6. TUNEL assay was used to detect apoptosis

The kidney tissue sections were fixed by PBS and restored with protease K working fluid. After the membrane was broken, the slides were fixed by PBS, and TdT was added after it was mixed with dUTP. The nucleus was restained with DAPI, and the film was sealed with an anti-fluorescence quenching agent after incubation at room temperature. For the fluorescence microscope observation, the FITC excitation wavelength was 465–495 nm, the emission wavelength was 515–555 nm, and apoptotic bodies were green.

### 2.3. Statistical analysis

SPSS19.0 software was used to process the data. Continuous variables with normal distributions were represented by mean value plus or minus the standard deviation. A paired sample *t* test was used for comparison between the two groups. Categorical variables were represented by percentage, and the Fisher exact probability method was used to compare the single-factor groups. *p* < .05 was considered statistically significant.

## 3. Results

### 3.1. Clinical indicators of the blood and urine of rats in each group

There were significant differences in the serum uric acid, kidney function and urinary protein in three groups of SD rats. In the second week, the creatinine and 24UTP in the HUA and GSH groups were higher than those in the NC group (*p* < .05); there was no statistically significant difference between the HUA and GSH groups (Table 1). The serum uric acid, creatinine and 24UTP in the HUA and GSH groups at the end of 4 weeks were higher than those in the NC group (*p* < .05). After the treatment of GSH, the serum creatinine and 24UTP were improved in the GSH group compared with the NC and HUA groups (*p* < .05) (Table 2). During the experiment, compared to the NC group, the serum uric acid of the HUA and GSH groups was increased. There was no statistically significant difference between the two groups.

**Table 1.** Blood uric acid, blood creatinine and urine protein analysis result in the 2nd week.

Group	Number	Blood uric acid (mmol L^−1^)	Blood creatinine (mmol L^−1^)	Urine protein (mg)
NC	10	113.33 ± 12.36^a^	44.20 ± 7.41^a^	5.01 ± 1.29^a^
HUA	10	284.83 ± 22.54^b^	101.60 ± 16.29^b^	33.44 ± 7.93^b^
GSH	10	279.09 ± 24.85^b^	96.10 ± 14.69^b^	29.38 ± 4.67^b^
*F*		218.21	56.133	82.12
*p*		0	0	0

$\bar{x}$±s , *n* = 10.

The blood uric acid, blood creatinine and urine protein in the HUA and GSH groups had increased compared with the NC group (^b^*p* < .05 vs. ^a^*p*, n = 10). The results showed that there was no statistical difference between the HUA and GSH groups. Which may be related to the increased blood uric acid caused kidney damage from the 2nd week.

**Table 2.** Blood uric acid, blood creatinine and urine protein analysis result in the 4th week.

Group	Number	Blood uric acid(mmol/L)	Blood creatinine(mmol/L)	Urine protein(mg)
NC	10	113.10 ± 15.60^a^	43.50 ± 8.78^a^	5.03 ± 1.05^a^
HUA	10	298.95 ± 27.46^b^	117.10 ± 18.25^b^	45.03 ± 9.89^b^
GSH	10	280.71 ± 30.08^b^	94.10 ± 16.25^c^	34.89 ± 4.75^c^
*F*		165.459	63.656	106.859
*P*		0	0	0

$\bar{x}$±s, *n* = 10.

The increased blood uric acid could cause kidney damage. In this study, we found that the blood uric acid, blood creatinine and urine protein of the HUA and GSH groups increased gradually compared with the NC group (^b,c^*p*< .05 vs. ^a^*p*, n = 10). At the end of the 4th week, the blood creatinine and urine protein of the GSH group decreased compared with that of the HUA group, which may be related to the effect of GSH (^c^*p* < .05 vs. ^b^*p*, n = 10).

### 3.2. Changes in MDA and SOD indicators in kidney tissues of rats in each group

At the end of 4 weeks, there was a significant difference in the concentrations of MDA and SOD in the kidney tissues. Compared to the NC and GSH groups, MDA concentrations in the HUA group increased, whereas SOD decreased (*p* < .05). MDA concentration in the GSH group increased compared to the NC group and decreased compared with HUA group, *p* < .05. SOD for the GSH group decreased compared with the NC group and increased in comparison to the HUA group, *p* < .05. The above results were statistically significant (Table 3).

**Table 3.** MDA and SOD analysis results (`x ± s, *n* = 10).

Group	Number	MDA	SOD
NC	10	0.75 ± 0.06^a^	63.89 ± 7.43^a^
HUA	10	2.41 ± 0.20^b^	42.97 ± 4.94^b^
GSH	10	1.72 ± 0.18^c^	52.95 ± 4.86^c^
*F*		280.23	31.808
*p*		0	0

In this study, we found that the MDA of the HUA and GSH groups increased compared with the NC group at the end of the 4th week, and the SOD decreased compared with the NC group (^b,c^*p* < .05 vs. ^a^*p*, n = 10). Compared with the HUA group, the MDA of the GSH group decreased and the SOD increased, which may be related to the effect of GSH (^c^*p* < .05 vs. ^b^*p*, n = 10).

### 3.3. Expression of p53, bax and caspase-9/-3 proteins in mitochondria

**Figure 1.**
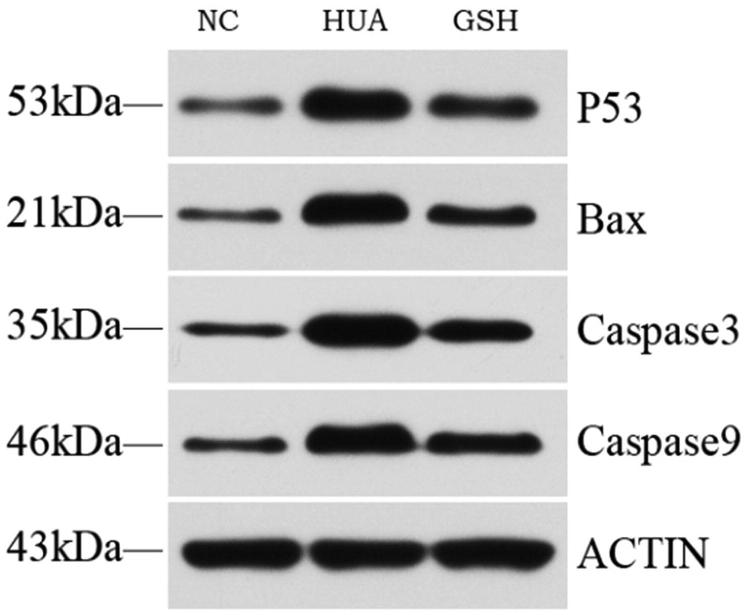
Detection the P53, Bax, Caspase3 and Caspase9 by western blot method.

**Figure 2.**
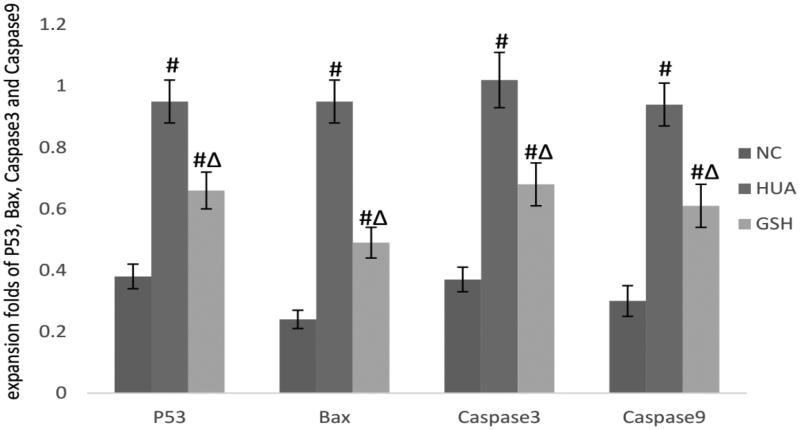
P53: *F* = 227.438, *p* = .000; compared with the NC group, #*p*_HUA_=.000, #*p*_GSH_=.000; compared with the HUA group, ^Δ^*p*_GSH_=.000. Bax: *F* = 492.754, *p* = .000; compared with the NC group, #*p*_HUA_=.000, #*p*_GSH_=.000; compared with the DN group, ^Δ^*p*_GSH_=.000. Caspase3: *F* = 210.801, *p* = .000; compared with the NC group, #*p*_HUA_=.000, #*p*_GSH_=.000; compared with the HUA group, Δ*p*_GSH_=.000. Caspase9: *F* = 238.550, *p* = .000; compared with the NC group, #*p*_HUA_=.000, #*p*_GSH_=.000; compared with the HUA group, Δ*p*_GSH_=.000.

The expression of p53, Bax, and Caspase-9/-3 protein was increased in the GSH group, but reduced in the HUA group. There were statistically significant differences between three groups (Figures 1 and 2).

### 3.4. Apoptosis of kidney tubular epithelial cells in three groups of rats was detected by TUNEL method

**Figure 3.**
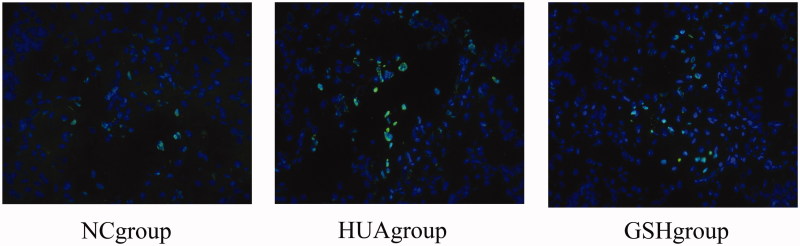
Apoptosis of kidney tubular epithelial cells in three groups of rats was detected by TUNEL method.

Analyzed with the immunofluorescence microscope, the apoptotic bodies were found in the nucleus of renal tubular epithelial cells from three groups. The number of apoptotic bodies in the HUA group was significantly higher than those in the NC and GSH groups. The number of apoptotic bodies in the GSH group was higher than that in NC group, but lower than that in HUA group. The Roche kit was labeled with FITC fluorescein, and the positive apoptotic nucleus was green (Figure 3).

## 4. Discussion

Seventy percent of uric acid in the human body is reabsorbed and secreted by the renal tubule. As observed in uric acid nephropathy animal models and pathological changes noted in the human kidney, uric acid kidney disease is mainly characterized by diffuse renal tubular interstitial lesions, renal artery sclerosis, and renal tubular epithelial cells damage. Therefore, renal tubular epithelial cells injury may be an important link of renal damage caused by HUA [8]. The changes in the structure and function of the renal tubule epithelial cells can lead to the uric acid excretion disorder, and other important functions of the renal tubules will also be damaged so as to lead to uric acid nephropathy.

In recent years, studies have confirmed that oxidation played an important role in the pathologic kidney injury. Some scholars copied the uric acid nephropathy rat model and observed HUA damage in rat renal tubular epithelial cells; these results suggested that the oxidative stress was the main mechanism through which uric acid caused the renal tubular epithelial cell damage [9]. During the occurrence and development of uric acid nephropathy, there was obvious apoptosis, cell proliferation, and cell differentiation in the renal tubular epithelial cells [10]. Under normal circumstances, the reactive oxygen species (ROS) that the body generated is cleared in a dynamic, equilibrium state, but when the body is in a state of oxidative stress, the oxidation and antioxidation gets out of balance. The intracellsular ROS would increase more than that the body could clear. This can lead to DNA oxidative damage, abnormal protein expression and tissue damage. MDA and SOD are often used as indicators of oxidative stress [11,12]. Increased MDA levels and decreased SOD activity can trigger an oxidative stress response, leading to the cell injury and even cell death. MDA and SOD are commonly used as the indicators to reflect the level of oxidative stress. The experiment used the uric acid nephropathy rat kidney medulla to study MDA, SOD and other indicators of oxidative stress found in the kidney medulla. MDA and SOD obviously changed in the HUA group, the concentration of MDA in the rat renal medulla increased significantly in the NC group, but SOD reduced significantly, tipping off uric acid nephropathy in rats by increasing oxidative stress reaction. This is one of the reasons behind renal tubular epithelial cells damage, and through the intervention of GSH, these changes improve the GSH groups’ MDA reduces HUA, and increases SOD. Additionally, it was suggested that GSH can reduce kidney damage by improving the oxidative stress response. Analyzed by the TUNEL method, it was observed that the rats in the NC group had a smaller number of renal tubular epithelial cell apoptotic bodies than the HUA group, whose small rat renal tubular epithelial cell apoptotic body number clearly increased. Moreover, the number of apoptotic bodies in the GSH group increased compared to the NC group but decreased in the HUA group. Prompt GSH administration improved oxidative stress and protected the renal tubular epithelial cells.

The mitochondrial damage that occurred during oxidative stress was the main damage deserved researched. After mitochondrial damage, a variety of protein would be expressed abnormally. Among them, the apoptosis protein was the focus of this study. In the mitochondria, p53 is an important factor in the oxidative stress sensitive signal distribution, nucleoli and intracellsular skeletal proteins. when cells are acted upon by a reactive oxygen species; DNA damage and stimulation, such as telomere erosion, p53 gene expression, protein expression, lower degradation, biological half-life, and obviously improve the stability and activity [13]. p53 can control the occurrence of apoptotic gene expression. On the one hand, p53 promotes the apoptotic protein expression, such as Bad, Bax, Bak, and so forth, and on the other hand, it reduces the expression of apoptotic protein [14], such as the Bcl-2, the Bcl-xl. The relative ratio of Bax/Bcl-2 determines the occurrence of apoptosis [15]. Bcl-2 and Bax, located in the cells membrane system, are a group of genes that regulate the cell apoptosis. Bcl-2 functions mainly to inhibit apoptosis, thereby to prolong cell life. Bax antagonizes the Bcl-2 apoptotic factor and is the representative of apoptotic gene expression levels that balance the common regulation of apoptosis [16]. In addition, p53 can further activate a Caspase cascade reaction and induce apoptosis by down-regulating the bcl-2 protein family [17]. Activated caspase-9 can activate caspase-3 to induce apoptosis. In this experiment, using western blot detection of p53, Bax, and Caspase-9/-3 related protein expression, p53 protein of the mitochondria in the HUA groups was affected by the oxidative stress response and showed increased expression. The higher expression of p53 regulated the expression of the downstream proteins including Bax and Caspase-9/-3. In the prosess, the expression of Bax, Caspase-9/-3 was increased; the apoptosis of renal tubular epithelial cells was increased; renal tubular function was impaired so as to cause the kidney disease. The important role of the p53-bcl-2/bax-caspase pathway in the whole pathogenesis was confirmed.

GSH is composed of glutamic acid, cysteine and glycine peptides, which exist primarily in the normal cells of the body. GSH has a variety of biochemical effects, for example, combining with a variety of chemicals and their metabolic products to remove oxygen ions and other free radicals, protecting the membrane, promoting the enzyme activity, and serving as an antioxidant. GSH is the main metabolic regulator of human body cell material [18]. This study performed uric acid nephropathy rat GSH intervention treatment and the results exhibited that the urinary protein, oxidative stress index, and the expression of apoptosis proteins, significantly improved in the GSH group, similar to previous research results. These findings showed that GSH can provide antioxidative protection against uric acid nephropathy in renal tubular epithelial cells and provide a powerful clinical tool for the treatment of uric acid nephropathy experimentally. Therefore, GSH has high scientific value and clinical significance.

## 5. Conclusion

Elevated serum uric acid levels can damage renal tubular epithelial cells through oxidative stress, increase epithelial cells apoptosis, and impair their structure and function. Mitochondria was the main organelles damaged in the epithelial cells via oxidative stress. Influenced by oxidative stress, the expression of the tumor suppressor gene p53 increased which led to the expression of the downstream apoptotic proteins including Bax, Caspase-9/-3. GSH can protect the renal tubule epithelial cells and improve renal tubule function by improving the response to oxidative stress.
